# Cell-free TXTL synthesis of infectious bacteriophage T4 in a single test tube reaction

**DOI:** 10.1093/synbio/ysy002

**Published:** 2018-01-22

**Authors:** Mark Rustad, Allen Eastlund, Paul Jardine, Vincent Noireaux

**Affiliations:** 1 School of Physics and Astronomy, University of Minnesota, Minneapolis, MN, USA; 2 Department of Diagnostic and Biological Sciences and Institute for Molecular Virology, University of Minnesota, Minneapolis, MN, USA

**Keywords:** cell-free transcription/translation, bacteriophages, self-assembly, cell-free synthetic biology

## Abstract

The bottom-up construction of biological entities from genetic information provides a broad range of opportunities to better understand fundamental processes within living cells, as well as holding great promise for the development of novel biomedical applications. Cell-free transcription–translation (TXTL) systems have become suitable platforms to tackle such topics because they recapitulate the process of gene expression. TXTL systems have advanced to where the *in vitro* construction of viable, complex, self-assembling deoxyribonucleic acid-programmed biological entities is now possible. Previously, we demonstrated the cell-free synthesis of three bacteriophages from their genomes: MS2, ΦX174, T7. In this work, we present the complete synthesis of the phage T4 from its 169-kbp genome in one-pot TXTL reactions. This achievement, for one of the largest coliphages, demonstrates the integration of complex gene regulation, metabolism and self-assembly, and brings the bottom-up synthesis of biological systems to a new level.

## 1. Introduction

The construction of complex biochemical systems in test tube reactions has become, in recent years, a thriving research area. Fostered by novel capabilities, deoxyribonucleic acid (DNA) assembly in particular, the bottom-up synthesis of genetically programmed biological systems *in vitro* has arisen as a means to quantitatively dissect the molecular mechanisms found in living cells (1, 2). Such an approach offers, at the same time, fresh perspectives for engineering active systems readily applicable to biotechnologies, biomanufacturing and medicine (3–6). Cell-free transcription–translation (TXTL) systems have emerged as amenable platforms for such realizations because they recapitulate the process of gene expression *in vitro*.

Whereas such systems were initially employed almost exclusively to produce protein outside living organisms, over the past 15 years cell-free TXTL has been transformed into a highly convenient technology for information-based constructive biology to address questions at both the basic and applied level (1, 7). The development of an all-*Escherichiacoli* TXTL toolbox, for example, has opened new perspectives for the synthesis of biochemical systems through the execution of natural or synthetic gene networks (8). The *in vitro* metabolism of this system, composed of an ATP regeneration system and a carbon source, fuels TXTL, yielding up to 2 mg/ml of protein synthesis in batch mode and up to 6 mg/ml in semi-continuous mode (9, 10), allowing the simultaneous expression of many genes. The transcription, based on the endogenous *E. coli* RNA polymerase and housekeeping sigma factor 70 (11), acts like a versatile operating system building on a vast repertoire of regulatory elements, as opposed to traditional TXTL platforms limited to a few bacteriophage RNA polymerases and promoters. This added complexity permits the execution of complex gene regulation pathways. This experimental platform has proven useful to prototype single genetic parts in isolation as well as circuit motifs (2, 12–14). The scalability of this system allows the completion of gene circuits from the femtoliter scale in cell-sized liposomes or microfluidics chips (15–18), to milliliters in test tube reactions.

**Figure 1.**
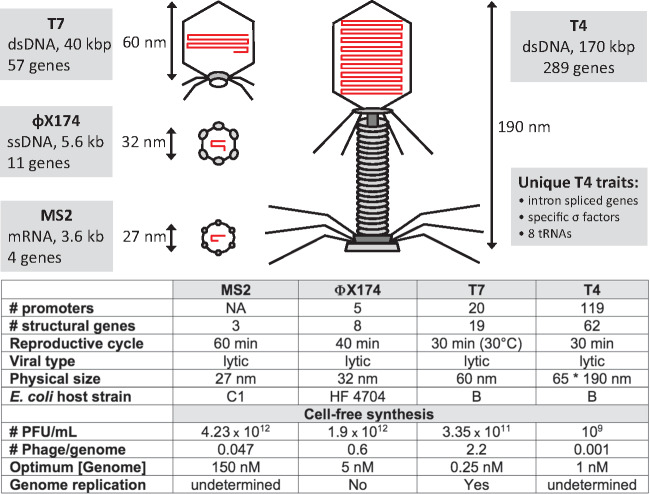
Overview of phages synthesized in TXTL. The synthesis of MS2, ΦX174 and T7 has been described previously (10, 19). Phage schematics have been drawn proportionally to size. The genomes shown in red are drawn proportionally to length. The table summarizes the characteristics of each phage and some of the optimum TXTL synthesis parameters for each of them. The number of structural genes is the number of gene products making the phage.

We recently demonstrated that this TXTL toolbox can process larger DNA programs than elementary circuit motifs. The bacteriophages MS2, ΦX174 and T7 are completely synthesized after a few hours by just adding viral genomes to a one-pot TXTL mixture (10, 19) (Figure 1). Surprisingly, the TXTL system was able to synthesize infectious T7 phages *in vitro*, concurrently with DNA replication, revealing unexpected potentials of cell-free protein synthesis. Together with MS2 and ΦX174, coliphages turned out to be a measure of the capabilities of TXTL to effectively achieve gene expression and complex self-assemblies into functioning wholes. In this work, we demonstrate that the TXTL toolbox can be challenged with the phage T4, one of the largest enterobacteriophages that infects *E. coli*. Remarkably, infectious T4 phages are produced in one-pot batch mode TXTL reactions using the same procedure used for the three other phages, by just adding the T4 genome to a standard TXTL reaction.

The coliphage T4 is among the largest and most complex viruses developed into a model system for the study of fundamental molecular events in biological systems, rivaling in size and complexity the eukaryotic herpesviruses. The T4 genome is comprised of 168 903 bp, encoding ∼289 genes (20). Whereas only 62 genes have been deemed essential to form viable T4 phages *in vivo*, many others support a wide array of functions including nucleoside metabolism, suppression of the host genome, encoding 8 tRNAs, and the timing of gene expression over the course of infection (21). A T4 phage particle is composed of more than 1500 proteins from about 50 different genes. It has been determined that 24 proteins are involved in the head assembly, 22 in the tail assembly, and 6 in formation of the tail fibers (22). More than 10 other genes not present in the assembled phages are necessary, including scaffolding and catalytic proteins, and chaperone-like proteins that assist polypeptide chain folding and protein assembly (20). The four virion parts (T4 head, tail, long tail fibers and whiskers) are assembled through four independent, linear pathways that converge to form a mature infectious phage (23). The completed T4 virion is 190 nm tall and 65 nm wide at its maximum. The complexity of gene regulation, metabolism, structure, and self-assembly found in T4 surpasses by far the ones found in the three other phages described above. T4 gene regulation includes overlapping genes, internal translation initiation, eukaryote-like introns spliced genes, translational bypassing, RNA processing, specific sigma- and anti-sigma factors, and its own DNA replication machinery (21). It is the degree of complexity found in T4 that positions it between the simplicity of a parasitic existence of a virus and the elaborate architecture of a free-living cell.

## 2. Materials and methods

### 2.1 Reagents

All chemicals were reagent-grade and purchased from Sigma Aldrich unless otherwise specified.

### 2.2 Phage T4 stock preparation

Phages were produced using a single-plaque, multi-cycle technique previously well described in Chen *et al.* (24). Briefly, *E. coli* B cells were grown in Luria-Bertani (LB) media in an overnight culture. This culture was diluted 50× into fresh LB media and a single 4–5 h wild-type T4 plaque grown on an LB agar plate with *E. coli* B cells was cored with a flame-sterilized Pasteur pipette and blown into the liquid culture. The culture was then allowed to incubate for 2 h and then monitored until the onset of superinfection. Superinfection was tested for by adding CHCl3 to a small volume of the infected cells and monitoring for lysis after 5 min. The cells were allowed to incubate for an additional 2 h before deoxyribonucleases (DNase) I (Sigma Aldrich) was added to a final concentration of 5 μg/ml. The solution was shaken at 37°C for an additional 5 min before the volume was then centrifuged at 10 000×*g* for 10 min at 4°C to pellet the infected cells. The supernatant was discarded and the pellet resuspended in 10 ml cold 1× TM [50 mM Tris (pH 7.8), 10 mM MgCl_2_] buffer containing 5 μg/ml DNase I. The concentrated cells were lysed with the addition of 200 µl of CHCl3 and heavy vortexing. The solution was centrifuged at 10 000×*g* for 10 min at 4°C to remove cellular lysis products. This stock was stored at 4°C over CHCl3 until a fraction was removed for further purification. The removed volume was purified by 5–45% (wt/vol) sucrose gradient and centrifuged at 35 000 rpm for 20 min in a swinging bucket rotor in an ultracentrifuge system. The bands were removed, re-diluted by 3× in 1× TM and re-centrifuged at 25 000 rpm for 1 h to pellet the phage. Each pellet was resuspended using 0.2 ml 1× TM buffer at 4°C overnight.

### 2.3 T4 genome extraction

Concentrated T4 stock was diluted 10× with Millipore H2O (Milli-Q Advantage A10) before adding an equal volume of saturated Tris: Phenol: Chloroform (ThermoFisher Scientific) solution to disrupt structural phage proteins. The layered solution was gently shaken by hand for 2 min until an emulsion was formed. This was immediately followed by 5 min of centrifugation at 4°C at 13 300 rpm. Due to the large size of the T4 genome, all following DNA handling steps were performed using wide-bore pipette tips to avoid shear damage. The aqueous phase was slowly pipetted off while avoiding disruption of the protein boundary. The pipetted volume was again mixed with an equal volume of Tris: Phenol: Chloroform and the process was repeated for a total of three phenol extractions. A final chloroform back extraction was performed to remove phenol from the genomic solution followed by 5 min of centrifugation at 4°C and 13 000 rpm before removing the aqueous phase to a fresh Eppendorf tube. This volume was then estimated and 2.8 volumes of 95% ethanol (EtOH) (ThermoFisher Scientific) were added for a total EtOH concentration of 70%. This was gently mixed and allowed to sit overnight at −20°C to precipitate DNA in solution. This solution was then centrifuged for 30 min at 13 300 rpm at 4°C to pellet the T4 DNA. The supernatant was discarded and 500 μl of 70% EtOH was added. The pellet was washed by flicking the tube and re-centrifuged at 13 000 rpm at 4°C, discarding the ethanol after. This step was repeated again for 2 total washes. The final pellet was allowed to air dry for 20 min at room temperature before resuspension of the DNA pellet in 100 μl of Millipore water. The final genomic concentration was determined using a NanoDrop One at 260 nm. Following genomic extraction, a fraction of T4 DNA was plated on agar with and without an *E. coli* B carpet to ensure that no plaques formed, indicating all phage were disrupted by the process or that there was no bacterial contamination, respectively.

### 2.4 TXTL system and T4 TXTL reactions

Preparation of the all-*E. coli* TXTL system (myTXTL, Arbor Biosciences) used in this work was described previously in several articles (11, 25). Transcription and translation are performed by the endogenous molecular components provided by an *E. coli* cytoplasmic extract, without addition of exogenous purified TXTL molecular components. TXTL reactions are composed of an energy buffer and 20 canonical amino acids solution (26). The energy buffer is composed of 50 mM Hepes pH 8, 1.5 mM ATP and GTP, 0.9 mM CTP and UTP, 0.2 mg/ml tRNA, 0.26 mM coenzyme A, 0.33 mM NAD, 0.75 mM cAMP, 0.068 mM folinic acid, 1 mM spermidine, 30 mM 3-PGA, either 10–15 mM maltose or 20–40 mM maltodextrin. A typical cell-free reaction is composed of 33% (v/v) of *E. coli* crude extract. The other 66% of the reaction volume is composed of the energy mixture, the amino acids and plasmids. The amino acid concentration was adjusted between 1.5 mM and 3 mM of each of the 20 amino acids. Mg-glutamate, K-glutamate and PEG8000 (polyethelyne glycol, average molecular weight of 8000) concentrations were adjusted based on the reaction test made, see text (typically 60 mM K-glutamate, 5 mM Mg-glutamate and 2% PEG8000 for P70a-deGFP, a reference plasmid for the TXTL system (10). Cell-free reactions are carried out in a volume of 5 µl to 20 µl at 29–30°C. The controls included two assays based on rifampicin, an inhibitor of the core RNA polymerase, and DNase I. Rifampicin was used at 100 μg/ml (122 µM) and DNase I at 1 µg/ml in TXTL reactions.

### 2.5 Plaque assay

The bacteriophage was counted using the standard plaque assay using the *E. coli* strain P301. The cells were grown in LB broth at 37°C, following a two-stage cascading culture protocol. Incubate a 5 ml LB pre-culture overnight to saturation; then, dilute the saturated pre-culture 50: 1 in 50 ml LB and grow host cells 3.5 h to mid-log phase. Centrifuge 50 ml culture for 10 min at 5000×*g* and resuspend in 5 ml LB. The plates were prepared as follows: each sample was added to a solution of 2.6 ml of 0.6% liquid LB-agar solution (45°C) and 0.025 ml of cell culture dispensed on a 1.5% solid LB-agar plate. Plates were incubated at 37°C for 7 h. The error bars shown in the plots are standard deviations of multiple repeats.

### 2.6 Electron microscopy

To visualize phages originating from the TXTL system, reactions were plated as above at concentrations high enough to completely lyse all cells on the plate. Phages were recovered from the plates by scraping the top agar from the plates into 2.5 ml 1× TM buffer. This extract was homogenized by vortexing and clarified by high speed centrifugation. The supernatant was then filtered through a 0.45 µm sterile filter, and phages were pelleted in a Ti-50.2 ultracentrifuge rotor at 30 000 rpm, 4°C for 90 min (Beckman Coulter). Pelleted phage were resuspended overnight at 4°C in 1× TM buffer. Phages were adsorbed to and imaged on carbon-coated formvar grids stained with 2% w/v uranyl acetate in a Tecnai Spirit TEM (FEI).

## 3. Results and discussion

**Figure 2.**
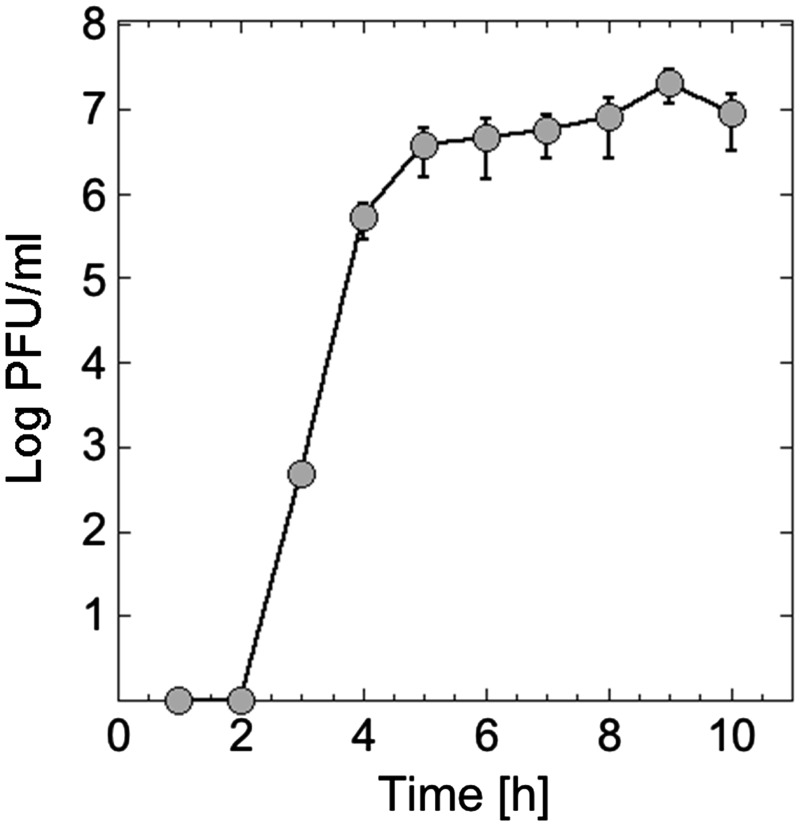
Kinetics of T4 TXTL synthesis. A concentration of 1 nM T4 genome and 3% PEG was used for this experiment. No phage is produced during the first 2 h of incubation. T4 synthesis reaches plateau within just a few hours, typically between 10^7^ and 10^9^.

The goal of the first experiment was to plot the kinetics of phage synthesis to determine the reaction incubation time and the potential yield of the system. Samples from a cell-free reaction set at the optimum biochemical settings (1 nM genome, 5 mM Mg, 60 mM K, 3% PEG8000) were plated at 1 h interval. No detectable phages were synthesized during the first 2 h. Most of the phages were synthesized between 2 and 4 h of incubation (Figure 2). Phage yield increases slightly after 4 h to plateau between 6 and 10 h. Although slightly slower than T7 (19), the T4 synthesis kinetics in TXTL presents the same trends as T7, with a burst of phages that lasts a few hours. Subsequently, all the TXTL reactions were incubated for 10–12 h.

**Figure 3.**
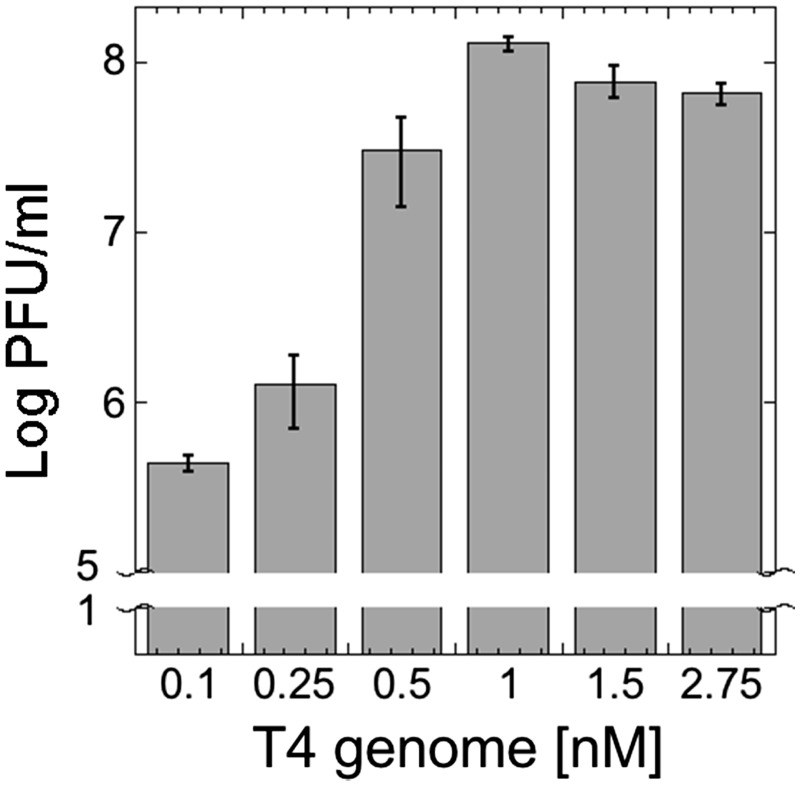
T4 TXTL synthesis as a function of genome concentration. Cell-free reactions with no genome added had no plaques. Standard TXTL reactions were carried out with 3% PEG. Reactions were incubated for 12 h. The optimum phage synthesis is observed at 1 nM genome.

Our next experiment consisted in varying the genome concentration in TXTL reaction. The goal was to probe the production capacity limit of the synthesis reaction, using the optimal reactions settings described above. Genome concentrations above the saturating limit are extraneous and genome concentrations below optimum do not fully exploit the assembly pathways and chemical energy present in the reaction. We observed that at 1 nM and above the reaction was saturated (Figure 3). Throughout the course of this work, T4 phage synthesis reached 10^9^ PFU/ml several times. It corresponds to about 1.66 × 10^−3^ phage produced per genome added to the reaction. We did not observe increased T4 synthesis when dNTPs were added to the cell-free reaction, suggesting that DNA replication does not occur. However, one limitation to address quantitatively this question was the high viscosity of the reaction due to the size of the T4 genome.

**Figure 4.**
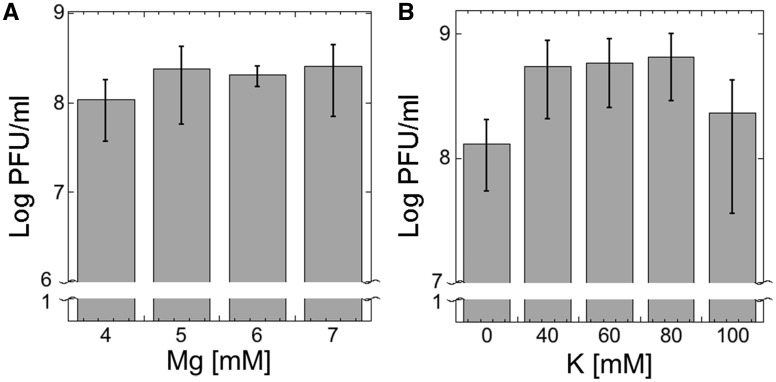
Magnesium and potassium settings for T4 TXTL synthesis. (**A**) Optimum phage synthesis is observed for a magnesium concentration of 4–7 mM. Phage yields are smaller outside this range. (**B**) 40–80 mM potassium is optimum for T4 TXTL synthesis.

In TXTL, some of the components show correlations to phage yield when their concentrations are varied, such as ion concentration and molecular crowders. We optimized the concentrations of magnesium, potassium, and PEG for T4 synthesis. Potassium glutamate (K-glu) and magnesium glutamate (Mg-glu) are essential ions for transcription and translation reactions and therefore highly important to be set at precise concentrations for T4 TXTL synthesis. Across a respective range of 0–100 mM K-glu and 4–7 mM Mg-glu, optimum concentrations for K-glu were found to be between 40 and 80 mM while there was no statistical difference between concentrations tested using Mg-glu (Figure 4). Outside of the 4–7 mM Mg-glu concentration range phage synthesis was notably smaller. The optimized concentrations that were used in reactions were 60 mM and 5 mM K-glu and Mg-glu, respectively.

**Figure 5.**
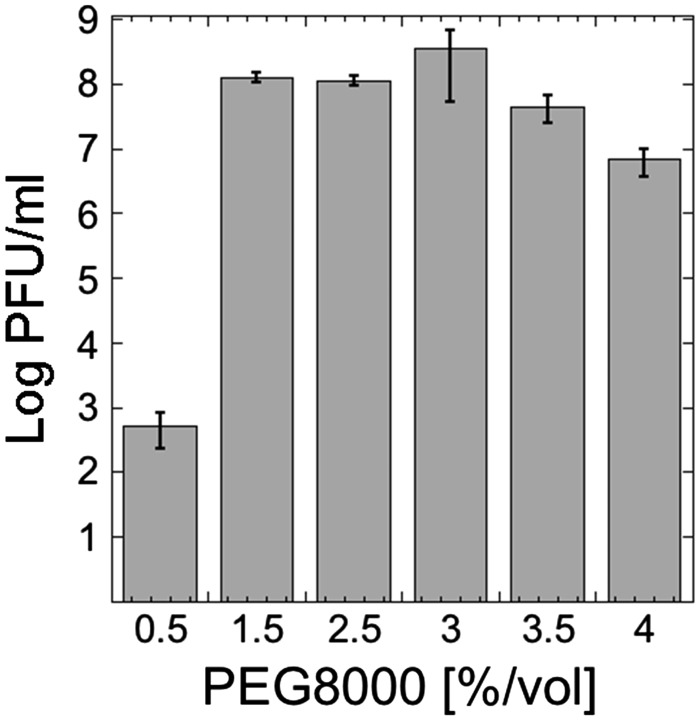
T4 TXTL synthesis as a function of PEG8000 concentration. Standard TXTL reactions were carried out with optimum settings: 5 mM magnesium, 60 mM potassium, 1 nM genome. Maximum TXTL T4 synthesis occurs around 3% PEG. The phage yields varies by orders of magnitude, as observed previously for the other phages ΦX174 and T7 (10).

It has been well established theoretically and experimentally that molecular crowding is a fundamental aspect of macromolecular assembly, especially in biological systems (27–29). Molecular crowding favors self-assembly by an entropy-driven process. In a solution containing large concentrations of macromolecules, such as a cytoplasm or an extract-based cell-free expression system, a net gain of entropy is created by decreasing the excluded volume when macromolecules self-assemble. It has been directly observed and modeled for the assembly of viruses such as HIV *in vitro* (30–32). Molecular crowding also alters the rates and the equilibrium constants of biochemical reactions by increasing the effective concentrations of macromolecules, promoting in particular the association of macromolecules (33, 34). Consequently, molecular crowding in TXTL reactions is an essential parameter to vary, especially when self-assembled systems are expressed. We already demonstrated the dramatic effect of PEG8000, a typical crowder for *in vitro* reactions, on the synthesis of the phage T7 in TXTL (10). In the case of T7, we showed that the standard molecular crowders settings in TXTL, 1.5–2% PEG8000, are not optimal when entire phages are expressed. By just increasing the PEG8000 concentration to 3–4%, we could increase TXTL T7 synthesis by a few orders of magnitude. We varied the concentration of PEG8000 (1% = 1.25 mM) in a TXTL reaction at optimal settings for T4 synthesis (1 nM T4 genome, 5 mM Mg-glu and 60 mM K-glu). The largest effect from any optimized parameter was the PEG concentration, showing a >100 000-fold PFU/ml increase when increasing from 0.5% to 1.5% v/v (Figure 5).

**Figure 6.**
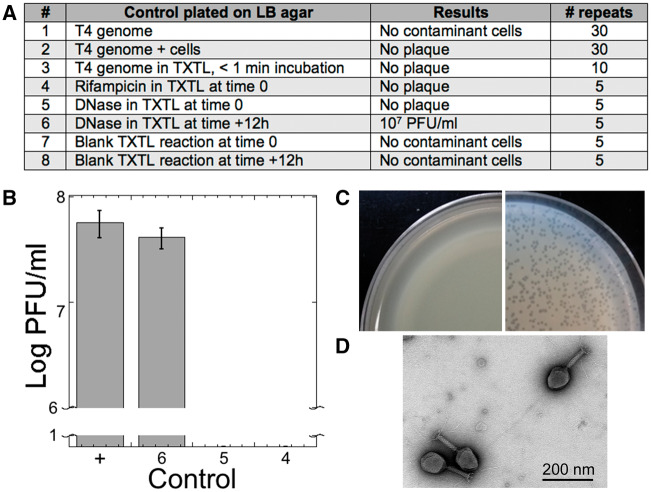
Experimental controls. (**A**) Table summarizing the controls and repeats. These controls were carried out during the course of experimentation. (**B**) Example of a set of controls including a positive (labeled as +, TXTL reaction containing 1 nM T4 genome) and controls 6, 5 and 4. (**C**) Example of control plates (negative on the left with a lawn of cells and positive on the right) for T4 TXTL synthesis. (**D**) Electron microscopy image of T4 phages from plaques produced by a TXTL reaction containing 1 nM of the T4 genome.

To eliminate any source of artifacts and contaminations, we performed eight types of controls during all stages of experimentation throughout the course of the work (Figure 6A), from genome extractions to cell-free reactions. Each of the T4 genome preparations used in this study was plated on LB agar multiple times to detect contaminating host cells (control 1). None were detected in the 30 repeats made. A similar control, done in parallel with control 1, was made by mixing the T4 genome with the *E. coli* host B (control 2) to test random T4 genome uptake by host cells. No plaque was formed in the 30 repeats. Control 1 and 2 covered all the T4 stocks prepared for this work. There were no observed plaques when the T4 genome was added to a cell-free reaction and incubated for <1 min (control 3) in the 10 performed repeats. When rifampicin (100 μg/ml or 122 µM), an inhibitor of the *E. coli* core RNA polymerase already tested in TXTL (10), was added to a cell-free reaction at time 0 with 1 nM T4 genome, no plaque was observed (control 4). When DNase I (1 µg/ml) was added to a cell-free reaction at time 0 with 1 nM T4 genome, no plaque was observed (Control 5). However, when DNase I was added to the cell-free reaction after 12 h of incubation and incubated for 30 min at 37°C, an average of 10^7^ plaque forming unit per milliliter (PFU/ml) was measured (control 6). In each of the final repeats, the equivalent of seven cell-free reactions of 12 µl (no DNA added, 84 µl total) was plated at 0 (Control 7) and 12 h (Control 8) incubation. No colonies were observed when plated showing that our cell-free expression system does not contain intact *E. coli* cells, as discussed previously (10). An example of the controls (Figure 6B) was performed as follows: a cell-free reaction containing 1 nM T4 genome was prepared and then split into equal volume reactions to which was added (i) water (positive control), (ii) rifampicin at time 0 (100 μg/ml or 122 µM), (iii) DNase I at time 0 (1 µg/ml) and (iv) DNase I added after 12 h of incubation. The plaque assay for T4 was carried out according to standard procedures (Figure 6C). We concluded that the synthesis of the phage T4 measured on plaques does not come from stock contamination. Despite multiple attempts, it was not possible to directly visualize the phage T4 from TXTL reactions by electron microscopy (EM) because of the high viscosity of the solutions and the relatively low number of phages synthesized (10^9^ PFU/ml at best). As an additional verification step, however, we visualized the phage T4 by EM from plaques produced from a TXTL reaction (Figure 6D), thus confirming cell-free synthesis of the phage T4.

## 4. Summary and conclusions

The objective of this letter is to report the synthesis of the largest biological entity so far achieved in a TXTL reaction. By demonstrating that TXTL systems can process a DNA program larger than 100 kbp encoding for about 100 essential genes, we bring the capabilities of the cell-free technology to a new level. We believe that the total synthesis of a phage of this size and complexity is a significant milestone towards the TXTL execution of a minimal cell genome for self-reproduction of a living entity. It has been estimated by different approaches and groups that the minimal set of genes required by a free-living cell is between 300 and 500 genes (35–38). The complete synthesis of T4 in an open environment like TXTL offers unique possibilities to interrogate quantitatively the links between gene expression, self-assembly and metabolism, which is left for a future study. Our future work will also consist of elucidating a few surprising results. For example, the literature suggests that the phage T4 initiates its self-assembly at the inner membrane of *E. coli* (20). It is unlikely to happen in TXTL because addition of liposomes prepared from *E. coli* membranes to TXTL reactions did not increase phage synthesis (not shown). This observation suggests that there are other thermodynamic modes that allow efficient self-assembly of complex biological systems in *in vitro* reactions lacking natural templates used for self-organization. The demonstration that MS2, ΦX174, T7 and now T4 can be entirely produced in TXTL reactions also paves the way toward phage engineering for biomedical applications. We expect that the all-*E. coli* TXTL platform can be employed to create synthetic phages, by recombineering for example, useful for phage therapy or other applications (39).

## Funding

The Office of Naval Research [N00014-13-1-0074]; the Human Frontier Science Program [RGP0037/2015]. Noireaux laboratory receives research funds from Arbor Biosciences, a distributor of myTXTL cell-free protein expression kit.

*Conflict of interest statement*. None declared.
